# Resistance of *Botrytis cinerea* to anilinopyrimidine fungicides: A novel ARMS‐PCR method for the detection of *Bcpos5* mutations and characterization of resistance using CRISPR/Cas9 editing

**DOI:** 10.1002/ps.70864

**Published:** 2026-04-28

**Authors:** Georgios Sofianos, Athanasios Petmezas, Anastasios Samaras, George Karaoglanidis

**Affiliations:** ^1^ Faculty of Agriculture, Forestry and Natural Environment, Laboratory of Plant Pathology Aristotle University of Thessaloniki Thessaloniki Greece; ^2^ Systemic Biology, Department of Organismal Biology Uppsala University Uppsala Sweden

**Keywords:** CRISPR/Cas9, cyprodinil, fungicide resistance, fungicide resistance detection methods, gene editing, gray mold, strawberry, tomato

## Abstract

**BACKGROUND:**

Anilinopyrimidine (AP) fungicides have been widely used against *Botrytis cinerea*, yet their resistance mechanisms have only recently been clarified. Resistance is primarily linked to mutations G408V, L412V, and L412F in the *Bcpos5* gene, whose encoded protein is localized to the mitochondria. In this study, we developed a detection method and tested the fitness of L412F/V mutants obtained by using the CRISPR/Cas9 editing technique.

**RESULTS:**

For rapid and cost‐effective mutation identification, a TETRA‐primer amplification refractory mutation system polymerase chain reaction (T‐ARMS‐PCR) was developed to rapidly detect the nucleotide alterations that lead to L412F and L412V mutations, producing a 702 bp band in all isolates, with additional 470 bp (F) or 252 bp (V) fragments. Isolates harboring only the 702 bp band were further digested with MlyI to confirm the mutation leading to the amino acid substitution G408V mutation (467 bp + 235 bp). Results were validated by Sanger sequencing. Application of the assay to 170 isolates from strawberry and tomato revealed mutation frequencies of 70.2% (L412F), 8.3% (L412V), and 4.7% (G408V) within the resistant fraction of the population (*n* = 82 resistant isolates). Furthermore, sequencing analysis revealed also a low frequency of the E407K mutation in *Bcmdl1*, along with evidence of additional, yet undefined, resistance mechanisms. To further characterize the mutations, the *B. cinerea* reference strain B05.10 was transformed with L412F and L412V alleles via CRISPR/Cas9 and homologous recombination. The resulting mutants displayed resistance to cyprodinil, and potential fitness costs were assessed in both field‐derived and CRISPR/Cas9‐generated isolates through measurements of mycelial growth and sporulation *in vitro*, and pathogenicity *in planta*. The L412F and L412V transformants did not differ significantly from the parental B05.10 strain in any of the evaluated fitness parameters.

**CONCLUSION:**

Overall, the developed ARMS PCR offers a fast, cost‐effective tool for resistance monitoring aiming to identify the most common mutations conferring resistance to APs, while CRISPR/Cas9 provides an efficient approach for functional validation of resistance mutations in *B. cinerea.* Using this approach, we confirmed that L412F and L412V mutations in *Bcpos5* confer resistance to APs, while are not associated with fitness cost. © 2026 The Author(s). *Pest Management Science* published by John Wiley & Sons Ltd on behalf of Society of Chemical Industry.

## Graphical abstract

A TETRA‐primer amplification refractory mutation system polymerase chain reaction (T‐ARMS‐PCR) method was developed for simultaneous detection of the L412F/V and G408V mutations. L412F/V mutants were created using the CRISPR/Cas tool and no fitness cost was observed.

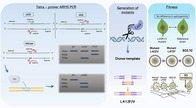

## 1 INTRODUCTION

*Botrytis cinerea*, the causal agent of gray mold disease, is a pervasive fungal plant pathogen that affects more than 1400 plant species, leading to significant agricultural losses worldwide.^1^ The management of this pathogen has been a critical focus in plant pathology with various fungicides having been developed to mitigate its impact. Among these, anilinopyrimidine (AP) fungicides have emerged as a vital tool in controlling *B. cinerea* infections.

Introduced in the early 1990s, AP fungicides, including pyrimethanil, cyprodinil, and mepanipyrim, have been widely adopted due to their strong activity against certain Ascomycete fungi and particularly against *B. cinerea* on vegetables, fruit, and ornamental plants.^2^ AP fungicides were initially believed to inhibit methionine biosynthesis.^3^ However, several studies' findings questioned this hypothesis^4^, ^5^ until Mosbach *et al*. reported that APs' primary target is of mitochondrial origin.^6^ Moreover, the same study demonstrated that the most frequent AP resistance‐conferring field mutations occur in two genes: the mitochondrial NADH kinase *Bcpos5*, harboring substitutions G408V/R and L412F/V, and the mitochondrial ABC transporter *Bcmdl1*, in which E407K was the predominant mutation, suggesting that one or both of these two genes could encode the molecular target.^6^

Regarding resistance development, AP fungicides are considered to be of medium risk according to the Fungicide Resistance Action Committee (FRAC). However, *B. cinerea* is a notorious high resistance risk pathogen with resistant isolates reported in field trials even prior to the commercial introduction of cyprodinil.^7^ This observation underscores the inherent adaptability of the pathogen and its propensity for rapid resistance evolution. Since the introduction of AP fungicides, resistant field isolates have been widely reported throughout the world on several crops.^8^, ^9^, ^10^, ^11^, ^12^, ^13^, ^14^, ^15^, ^16^

Most traditional methods of detection and monitoring of fungicide resistance include phenotypical bioassays and molecular techniques. Phenotypical bioassays rely on calculating the half‐maximal effective concentration (EC_50_) or the minimum inhibitory concentration (MIC). Molecular techniques include gene sequencing; however, this method usually leads to significant delays and extra costs. A solution to this issue can be the TETRA‐primer amplification refractory mutation system polymerase chain reaction (T‐ARMS‐PCR), which is a powerful and cost‐effective technique that can be efficiently used for genotyping single nucleotide polymorphisms (SNPs).^17^ It is based on the employment of four different primers in a single reaction. Initially, the two inner primers are designed to specifically bind to the alleles of interest, while the two outer primers amplify the larger fragment and assist in the generation of each PCR product.^18^ It only requires standard PCR reagents and equipment, followed by gel electrophoresis for the separation and visualization of the PCR products, significantly reducing time and cost associated with genotyping.^19^, ^20^

The development of fungicide resistance is often associated with fitness costs, which are the trade‐offs resistant strains face, compared to their sensitive counterparts. This phenomenon occurs due to genetic mutations in target genes that provide an edge when some kind of selection pressure (i.e., fungicides) is constantly applied. At the same time, they lead to the reduced ability of the resistant fungi to survive and reproduce compared to their sensitive populations in a non‐selective environment.^21^ Such effects may also result from resistance‐associated mutations that impair the functional efficiency of their molecular targets, thereby imposing fitness costs on the organism. In *B. cinerea*, fitness costs have been associated with fungicide resistance.^22^, ^23^, ^24^ However, studies have shown that AP resistance is not directly linked to fitness defects.^25^ A possible explanation could be the fact that most traditional methods to study fitness cost involve field strains. The possibility of fitness costs cannot be excluded, as fitness comparisons are often conducted using field strains with diverse and largely unknown genetic backgrounds. Thus, the occurrence of compensatory mechanisms circumventing fitness costs cannot be ruled out. To minimize the influence of additional, uncharacterized mechanisms, recent studies have effectively used transformed strains to assess fitness effects directly attributable to specific resistance mutations.^26^, ^27^ As a result, the use of generated mutants with site‐directed alterations becomes a necessity.

The CRISPR/Cas (clustered regularly interspaced short palindromic repeats/CRISPR‐associated proteins) system has become a groundbreaking technology in the field of genetic engineering. Originally discovered as a part of the adaptive immune system in bacteria and archaea, CRISPR/Cas9 has been adapted by scientists into a powerful tool for precise genetic editing.^28^, ^29^ The system consists of Cas9 endonuclease and a single‐guide RNA (sgRNA). The sgRNA sequence, approximately 20 nucleotides long, aligns with the target site for editing, based on complementarity. This site is adjacent to a unique protospacer‐adjacent motif (PAM) region specific (NGG) to Cas9. This alignment guides Cas9 to the precise editing location, leading to the creation of a double‐stranded break (DSB) in the DNA.^30^ The CRISPR/Cas system needs to be delivered into the genome to perform the DSB and this can be carried out through plasmid delivery or as a free ribonucleoprotein (RNP). The latter seems to provide significant advantages over plasmid delivery, such as not incorporating into the genome.^31^ After undergoing a DSB, the organism must repair this potentially life‐threatening damage using two major DNA repair pathways: non‐homologous end joining (NHEJ) and homologous recombination (HR). In knock‐in experiments, DNA homologous to the flanking sequences of the DSB is provided to enhance the homology‐directed repair (HDR). The donor template can possess alterations compared to the wild‐type (WT) sequence such as insertions, deletions or single nucleotide modifications.^32^ CRISPR/Cas technology has been effectively utilized in a range of fungal species^33^ and recently Leisen *et al*.^34^ developed a similar protocol for genome engineering in *B. cinerea*. These recent discoveries facilitate the detailed study of mutations and their individual impacts on the biological characteristics of fungi.

In our study, we developed an efficient, rapid, and cost‐effective tool for the simultaneous detection of the three most common AP resistance‐conferring mutations in *Bcpos5* (L412F, L412V, and G408V). The method utilizes T‐ARMS PCR to detect L412F and L412V in a single reaction, followed by digestion using the restriction enzyme MlyI to effectively identify the presence of G408V based on Restriction Fragment Length Polymorphism (RFLP). This entire procedure is conducted in a single tube, significantly reducing costs and time delays. Additionally, we investigated potential fitness costs associated with these mutations. We successfully created L412F and L412V mutants using the CRISPR/Cas system and compared them to field strains and to reference strain B05.10, aiming to assess differences in fungicide sensitivity, mycelial growth, sporulation, and virulence. The results of this study aim to facilitate the rapid detection of AP resistance in the field and provide insights into the fitness components of resistant populations, potentially leading to improved resistance management strategies.

## 2 MATERIALS AND METHODS

### 2.1 Fungal isolates

The *Bcpos5* mutations detection method was designed and tested on *B. cinerea* isolates collected and characterized for the requirements of a previous study.^16^ In total, 25 single‐spore isolates were used for the development of the method comprising five distinct group of isolates: (i) isolates with the L412F mutation (five isolates), (ii) isolates with the L412V mutation (five isolates), (iii) isolates with the G408V mutation (five isolates), (iv) AP‐resistant isolates lacking these mutations (five isolates), and (v) AP‐sensitive isolates (five isolates). These isolates were used exclusively for the method development.

For the validation and evaluation of the method, 60 isolates collected from three different tomato glasshouses in Crete (Greece) and 110 isolates collected from six different strawberry fields in Manolada (Greece) were used. The isolates were collected by scraping the spores from rotten host tissue using a sterile cotton swab, transferring them onto Petri dishes containing acidified potato dextrose agar (PDA; Neogen, Lansing, MI, USA). PDA was acidified after autoclaving by the addition of sterile 10% lactic acid to a final pH of approximately pH 3.8. Plates were incubated at 20 °C for 24 h. Then, single germinated conidia were observed under dissecting microscope and transferred to fresh PDA dishes for growth at 20 °C for 3 days. The collected single spore isolates were maintained at 4 °C until use.

For the characterization of the CRISPR/Cas9 generated mutants in terms of sensitivity to AP fungicides and their fitness characteristics, five CRISPR/Cas9‐generated L412F mutants and five L412V mutants were used. For comparison purposes, in addition to the CRISPR/Cas9 generated mutants, ten AP‐resistant field isolates, five AP‐sensitive field isolates and the reference sensitive strain B05.10 were included in the study. Of the ten AP‐resistant field isolates, five were possessing the L412F mutation and five were possessing the L412V mutation.

The reference strain B05.10 was used as a control sensitive isolate during all the experiments, while it was also used for the transformation protocol.

### 2.2 Measurement of sensitivity to APs

For the AP‐resistance screening, all the 170 isolates collected from tomato and strawberry fields were tested in a discriminatory dose sensitivity assay by transferring mycelial plugs excised from the margins of 4‐day‐old fungal colonies grown on PDA to plates containing Gamborg B5 medium (3 g of Gamborg, 1.36 g of potassium dihydrogen phosphate (KH_2_PO_4_), 9.9 g of glucose, and 15 g of agar per liter of medium, pH 5.5) amended with 7 ppm cyprodinil (Chorus 50 WG; Syngenta Hellas, Inofyta, Greece),^13^ and then incubated at 20 °C in the dark for 3 days. After 72 h of incubation at 20 °C in the dark, mycelial growth was evaluated. Isolates showing clear, visible mycelial growth were classified as resistant, whereas isolates displaying no growth were classified as sensitive to AP fungicides.^16^

To assess sensitivity to AP fungicides of the CRISPR/Cas9 generated mutants as well as the selected field AP‐resistant and sensitive isolates, EC_50_ measurements were conducted by measuring mycelial growth inhibition in plates containing Gamborg B5 medium amended with 0, 1, 2.5, 5, 7.5, 10 and 15 μg mL^−1^ cyprodinil for the resistant strains and 0, 0.01, 0.05, 0.1, 0.5, and 1 μg mL^−1^ for the sensitive ones, after 3 days. The experiment was conducted three times, with three technical replicates per isolate in each trial.

### 2.3 Primer design and TETRA‐primer ARMS PCR method

**Figure 1:**
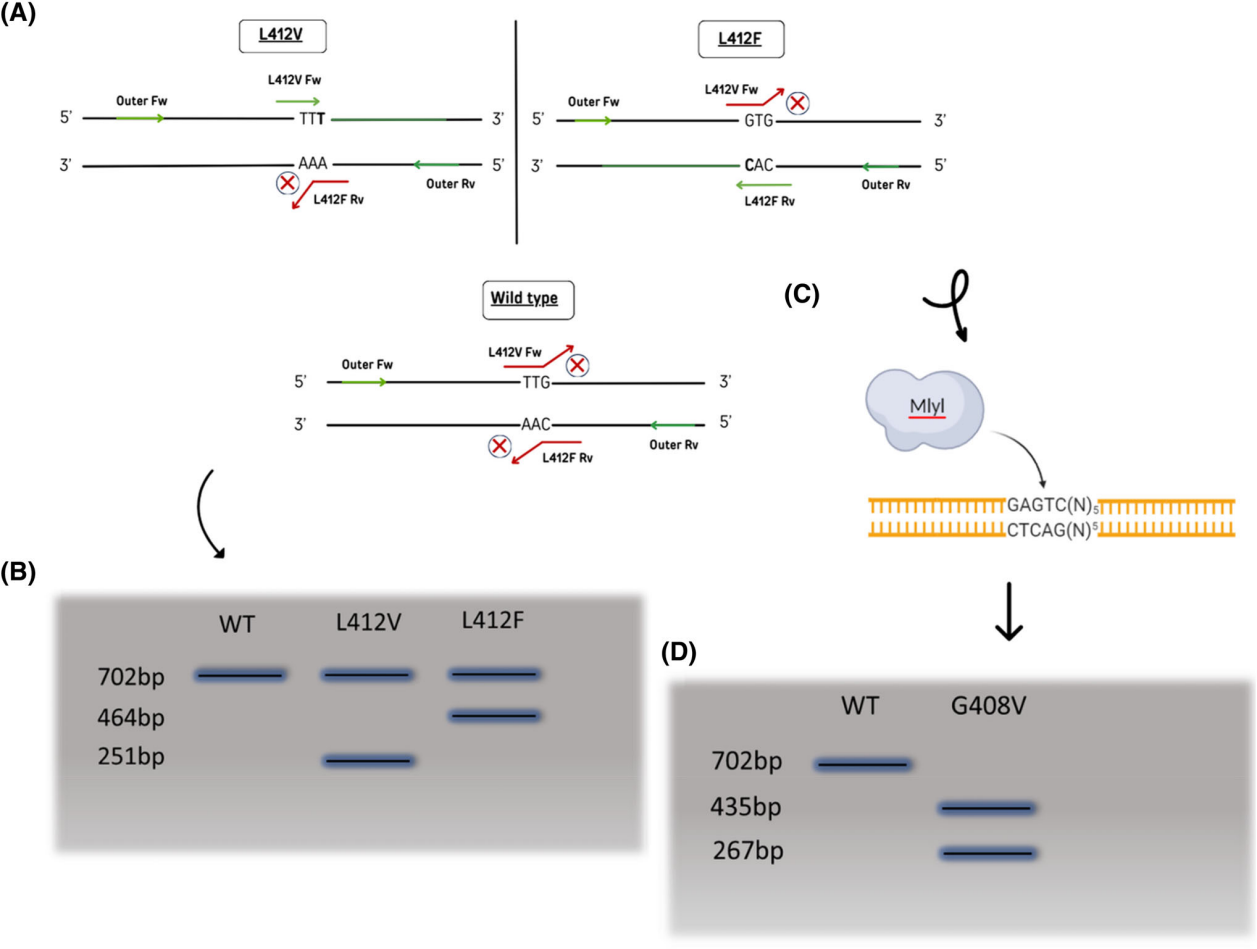
Schematic design of the procedure of the T‐ARMS PCR followed by digestion with Mlyl. (A) The T‐ARMS PCR consists of two outer primer and two allele‐specific ones. The binding sites of the primers are depicted on the gene *Bcpos5*. Non‐specific binding of the inner primers is inhibited by using deliberate mismatches in the sequence of the inner primers. (B) Electrophoresis showing the generated PCR products and their lengths allowing the easy detection of the L412F and L412V mutations. All genotypes yield a 702 bp band generated from the outer primers while samples possessing the L412F or L412V mutations yield an additional 464 bp or 252 bp band, respectively. (C) PCR products showing only the 702 bp band were further digested by Mlyl for the detection of the G408V mutation. (D) Samples possessing the G408V substitution were successfully digested by Mlyl yielding two distinctive bands of 435 bp and 267 bp.

The ARMS PCR assay was designed to detect L412F and L412V which are the two most common AP resistance mutations in *Bcpos5*. The reaction included four different primers, two outer and two inner ones (Table 1). Each inner primer was designed to specifically bind to DNA containing the desired mutation.^17^ In order to avoid unspecific binding, one extra deliberate mismatch was added in the inner primers' sequence, proximal to the 3′ end. The deliberated mismatches were carefully introduced so that a weak mismatch was followed by a strong one and vice versa.^35^, ^36^ The outer primers were positioned at asymmetrical distances from the mutation site, allowing for the generation of PCR products that are easily distinguishable on an agarose gel based on their different length (Fig. 1(A)).^37^ A gradient PCR was conducted for the determination of the ideal annealing temperature (data not shown). The final optimized PCR was carried out in tubes of 25 μL reaction volume containing: 12.5 μL PCR TaqNova‐RED (BLIRT, Qiagen Gdańsk, Gdańsk, Poland), outer primers at a final concentration of 0.3 μm each, inner primers at a final concentration 0.2 μm each, 100 ng of genomic DNA and molecular water to 25 μL. Thermocycling conditions were as follows: 95 °C for 3 min followed by 32 cycles of denaturation at 95 °C for 30 s, annealing at 58 °C for 35 s, extension at 72 °C for 20 s and final extension at 72 °C for 2 min. The separation of the PCR products was conducted by electrophoresis on 2% agarose gel stained with Midori Green Advance (NIPPON Genetics EUROPE, Duren, Germany) (Fig. 1(B)). The T‐ARMS PCR method was applied to all 170 isolates collected for the validation of the method. Genomic DNA extraction was performed from freeze‐dried fungal tissue as previously described.^38^

**Table 1:** Primers' sequence and polymerase chain reaction (PCR) product size for TETRA‐primer amplification refractory mutation system (ARMS) PCR

Primer	Sequence 5′‐3′	Primer length (bp)	GC content (%)	Product size (bp)	Temperature (°C)
BcPos_Beg	AGCACATTTAGCCATTATTGAAGTTT	26	30.70	702	53.9
BcPos_End	TCTGGGGCTGCTAGAAACA	20	50		55.9
V_Fw	ACGGTTGGGTTGGAGGCTTGAATGG**A** G	27	75	251	64.4
F_rev	CAAAAGGATGGTTGAACTTCA**G** A	23	39.10	464	53.4

*Note*: Bold letters correspond to extra deliberate mismatches; underlined letters correspond to mutation‐specific nucleotide.

### 2.4 Digestion with MlyI for detection of G408V

To detect the third most common AP‐resistance conferring mutation in *Bcpos5* in Greek populations, that leads to the amino acid change G408V, PCR products from the aforementioned reaction were digested by the restriction enzyme MlyI (New England Biolabs (NEB), Beverly, MA, USA) (Fig. 1(C)). More specifically, PCR products which resulted only from the binding of the outer primers and thus yielded a single 702 bp band were further digested by MlyI as per the supplier's instructions: 10 μL of PCR product, 5 μL (1×) 10× rCutSmart Buffer, 1 μL (10 units) of MlyI and nuclease‐free water to a total volume of 50 μL followed by incubation at 37 °C for 15 min and subsequent electrophoresis on 2% agarose gel (Fig. 1(D)).

### 2.5 Validation of the detection PCR tool and digestion assays

To assess the accuracy and efficiency of the assays, PCR products (*n* = 60) were also subjected to Sanger sequencing and the results were compared to those of both T‐ARMS PCR and enzymatic digestion. The outer primers (Bcpos_Beg/Bcpos_End) were also used for validation sequencing (Table 1) along with the primers Bcpos F/F2/R/R2 enabling coverage of most of the *pos5* gene.^16^ Primers used for Sanger sequencing of *Bcmdl1* gene were MDL_Fw/MDL_Rv (Supporting Information, Table S1).

### 2.6 Generation of L412F/V mutants

#### 2.6.1 *In vitro* sgRNA and donor template synthesis and RNP formation

Design and appropriate selection of sgRNA was conducted by using the CHOPCHOP design tool.^39^ The sgRNA was synthesized from DNA oligonucleotides, containing the T7 promoter sequence, 3 Gs (guanines), the desired protospacer sequence and the constant region. Synthesis of donor template was also carried out by using DNA oligonucleotides containing the desired mutation, TTG to TTT for L412F and TTG to GTG for L412V, along with a silent mutation in the PAM region, to potentially prevent the Cas protein from re‐cutting the same region after successful incorporation of the desired mutation (Table S1). In both cases, 10 μmol of the oligos were mixed to a final volume of 10 μL and then annealed at 95 °C for 5 min, from 95 °C to 85 °C at 2 °C s^−1^, from 85 °C to 25 °C at 0.1 °C s^−1^. Afterwards, T4 DNA polymerase (NEB) was used to fill‐in and extend the already annealed oligonucleotides as per the following reaction: 2.5 μL 10 mm dNTPs, 2 μL 10× NEB buffer 2.1, 5 μL nuclease‐free water and 0.5 μL enzyme followed by incubation at 12 °C for 20 min and purification by using the DNA Clean & Concentrator‐25 kit (Zymo Research, Irvine, CA, USA). While the donor templates were ready to use, sgRNA synthesis continued by using T7 RNA polymerase (NEB) in the following reaction and conditions: 6 μL 25 μM NTPs, 1.5 μL T7 buffer, 1.5 μL T7 polymerase, 2 μg DNA template and nuclease‐free water up to 20 μL followed by incubation at 37 °C overnight. Afterwards, DNase treatment was carried out according to the manufacturer's protocol: 14 μL water, 4 μL DNase I buffer (10×) and 2 μL DNase I (NEB) were added to the already transcribed RNA 20 μL reaction and further incubated at 37 °C for 15 min. RNA purification was carried out by using the RNA Clean & Concentrator‐25 kit (Zymo Research) and the amount and quality of produced RNA was determined by using P330 spectrophotometer (Implen GmbH, Munich, Germany). Cas9‐NLS (EnGen® Spy Cas9 NLS) was purchased from NEB. RNP formation was conducted by mixing 2 μL Cas9, 1.5 μL NEB 3.1 buffer, 2 μg sgRNA and water up to 15 μL, then incubating at 37 °C for 30 min.

#### 2.6.2 Transformation of *B. cinerea*

The model strain B05.10 was used as a parental strain for the transformation procedure. The transformation protocol was based on a PEG‐mediated protoplast approach according to previously published studies.^34^, ^40^, ^41^ The same sgRNA was used for both L412F and L412V mutant generation while one distinct repair template corresponding to each mutation was used (Table S1). For the direct selection of transformants, protoplasts were immersed in 50 mL liquid (42 °C) SH agar (0.6 m sucrose, 5 mm Tris–HCl pH 6.5, 1 mm ammonium dihydrogen phosphate (NH_4_)H_2_PO_4_, 9 g L^−1^ bacto agar), amended with 5 μg ml^−1^ cyprodinil. Positive colonies were sub‐cultured into plates containing the same concentration of cyprodinil, for four rounds to ensure pure lines devoid of WT nuclei. To verify the successful integration of the donor template along with the desired resistance‐conferring mutation and the silent PAM mutation, DNA from the mutants was sequenced by using the primer pair (Bcpos_Beg/Bcpos_End) (Table 1) for both PCR and sequencing.

### 2.7 Fitness measurements of CRISPR/Cas9‐generated mutants and reference isolates

#### 2.7.1 *In vitro* mycelial growth and sporulation

Mycelial plugs of 5 mm in diameter, obtained from fresh fungal colonies were transferred to the center of plates containing PDA medium. The isolates were incubated at 20 °C in the dark, and 5 days later colony diameter was measured. For the sporulation assays, fungal cultures were cultured on PDA Petri dishes under continuous light for 12 days to allow abundant sporulation. Afterwards, conidial suspension was produced by scratching the fungal surface with the aid of water and a sterilized scalpel followed by filtration through miracloth. The spores were counted using a Neubauer hemocytometer (Heinz Herenz, Hamburg, Germany) under a light microscope (Axio Lab.A1; Carl Zeiss AG, Jena, Germany). Both experiments were conducted three times using independent biological material with three technical replicates per isolate in each trial.

#### 2.7.2 Pathogenicity on bean plants

Pathogenicity tests were performed on leaves of seedling bean plants (*Phaseolus vulgaris* cv. ‘Malibu’), at the stage of two true leaves. Conidial suspensions of 10^5^ mL^−1^ spores in Gamborg medium broth were used as inoculum.^42^ Briefly, 10 μL of the suspension were carefully placed on the adaxial lamina of the leaf with the aid of a pipette. Three bean plants were used, thus six leaves, per isolate, were artificially inoculated. The artificially inoculated plants were kept in an incubation chamber at 22 °C and 100% humidity, generated by an air humidifier, for 5 days. Subsequently, disease severity was calculated by measuring the lesion diameter on the leaves. The experiment was conducted three times using independent biological material.

### 2.8 Statistical analysis

For the statistical analyses and graphical design, the GraphPad Prism software was used. Data from fungicide sensitivity, fitness and pathogenicity assays were tested for normality using D'Agostino and Pearson test (*α* = 0.05) followed by one‐way analysis of variance (ANOVA) assuming Gaussian distribution. *Post hoc* analysis was conducted based on Tukey's test (*α* = 0.05). The EC_50_ values were determined by plotting the relative inhibition of the mycelial growth against the log_10_ of fungicide concentrations. Mean EC_50_ values are presented as geometric means of independent biological replicates. Calculations were performed with SAS (JMP; SAS Institute, Cary, NC, USA).

## 3 RESULTS

### 3.1 Frequency of AP resistance and application of T‐ARMS PCR and MlyI digestion for detection of L412F/V and G408V mutations in *Bcpos5*

**Figure 2:**
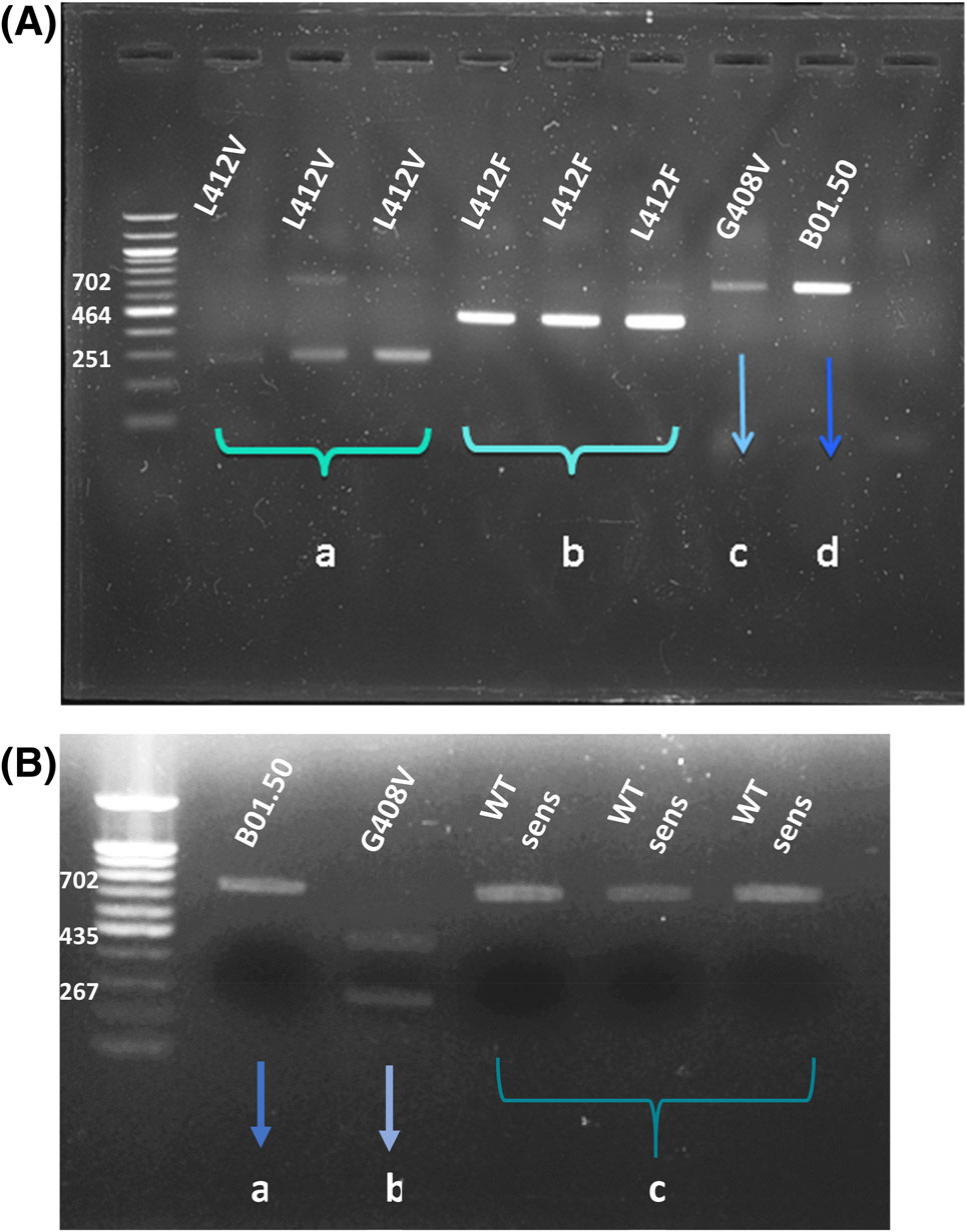
T‐ARMS‐PCR followed by digestion by Mlyl for *pos5* mutations identification from *Botrytis cinerea* isolates. (A) (a) amplicons of 251 bp and 702 bp indicate the presence of L412V, (b) amplicons of 464 bp and 702 bp indicate the presence of L412F, (c) amplicon of 702 bp originating from a strain carrying G408V, (d) amplicon of 702 bp originating from WT (B05.10). (B) Digestion products originating from samples that yielded a single 702 bp band in the T‐ARMS PCR. (a) B05.10, (b) field sample possessing the G408V mutation in *Bcpos5* gene effectively digested by Mlyl and showed two distinct bands of 435 bp and 267 bp, (c) sensitive field samples possessing no mutations in *Bcpos5* gene.

**Figure 3:**
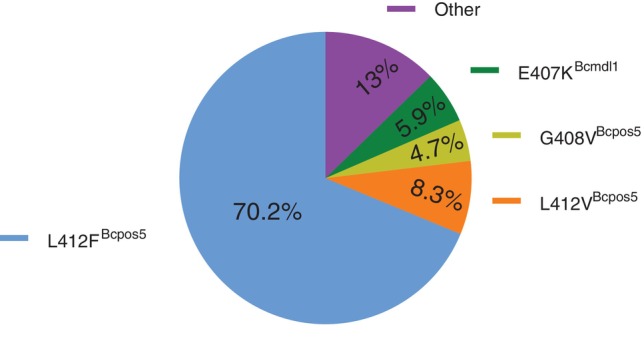
Pie chart showing the frequency distribution of AP resistance‐associated mutations detected in field samples (*N* = 82). Mutations were identified using T‐ARMS PCR for L412F/V, MlyI restriction digestion for G408V, and Sanger sequencing for E407K.

Of the 170 *B. cinerea* isolates tested, 82 were phenotypically characterized as AP‐resistant. The T‐ARMS PCR tool was applied to the entirety of the isolates, both sensitive and resistant, to better assess the risk of false positive results. Isolates that possessed neither L412F/V nor G408V mutations yielded a single 702 bp band. Isolates possessing the L412F mutation yielded a 464 bp band and a 702 bp band while isolates with the L412V mutation produced a 251 bp band and the 702 bp band (Fig. 2(A)). The absence of the 702 bp band produced by the outer primer binding in isolates possessing an L412 substitution was a common phenomenon (occurring at a frequency of almost 70%), however, this could be attributed to inner‐outer primer competition and it did not affect the detection of L412F and L412V genotypes. Consequently, PCR products yielding only a single 702 bp band were subjected to digestion by MlyI with DNA containing the G408V mutation yielding two distinct bands of 267 bp and 435 bp (Fig. 2(B)). Out of 170 isolates, all 88 sensitive isolates produced no mutation‐specific band, further validating the specificity of the developed detection tools. Regarding the 82 resistant ones, 56 of the isolates (70.2%) possessed the L412F mutation, followed by six isolates (8.3%) and four isolates (4.7%) for mutations L412V and G408V respectively (Fig. 3).

Because the source of AP‐resistance of 16 phenotypically resistant isolates was still unclear, their *Bcmdl* gene was sequenced. Five of them were found harboring the E407K mutation, while the remaining 11 isolates yielded no substitutions in either *Bcpos5* or *Bcmdl1* gene after having these two genes sequenced, implying the existence of additional AP‐resistance loci (Fig. 3).

### 3.2 Fungal transformation, selection and validation of knock‐in mutants

The transformation of the model *B. cinerea* strain B05.10 was based on the PEG‐mediated approach and the CRISPR/Cas editing tool was used to generate a DNA DSB to promote the homologous recombination repair pathway. The efficiency of the designed sgRNAs was calculated by the CHOPCHOP tool to achieve both high efficiency values and proximity of the generated DSB to the knock‐in site. Transformants were directly selected by the use of 5 μg mL^−1^ cyprodinil and then consecutively sub‐cultured four times to achieve homokaryosis. Successful incorporation of L412F/V and the silent PAM mutation were validated by direct sequencing of positive colonies.

### 3.3 Comparison of AP sensitivity of field and knock‐in mutant strains

**Figure 4:**
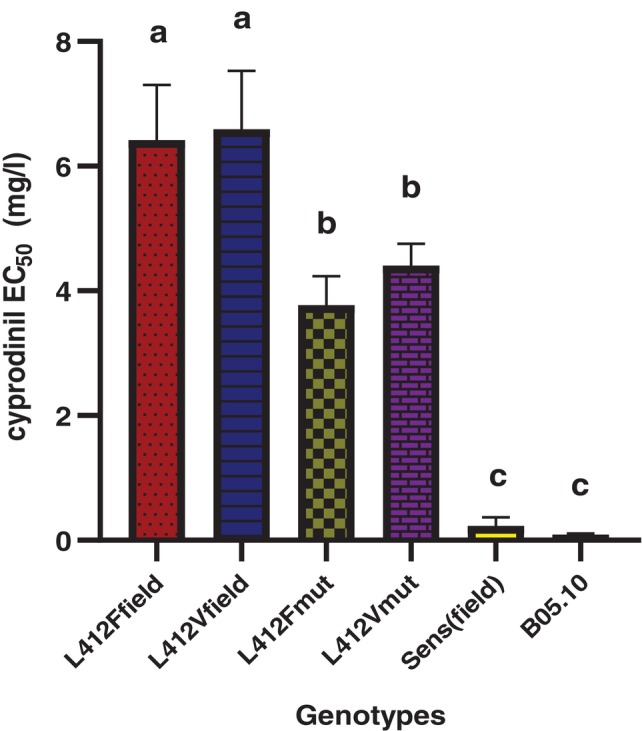
Sensitivity of *Botrytis cinerea* isolates with different genotypes to cyprodinil measured as EC_50_ values (μg mL^−1^). L412Ffield and L412Vfield refer to field isolates possessing the mutations, while L412Fmut and L412Vmut refer to mutants generated from B05.10 strain. Sens(field) refers to AP‐sensitive field strains. Five isolates were used for each genotype, with three technical replicates each. The experiment was conducted three times. Different letters represent statistically significant differences based on Tukey's test (*α* = 0.05).

Successfully generated L412F/V mutants showed resistance to cyprodinil, with mean EC_50_ values of 3.63 μg mL^−1^ and 4.12 μg mL^−1^, respectively, while B05.10 showed a mean EC_50_ of 0.09 μg mL^−1^ and sensitive field isolates showed a mean of 1.46 μg mL^−1^. L412V mutants showed higher resistance levels than L412F ones but their difference was not found to be statistically significant. However, field strains showed significantly reduced sensitivity to cyprodinil compared to mutants possessing the corresponding mutations with their EC_50_ values exceeding 6 μg mL^−1^ (Fig. 4).

### 3.4 Mycelial growth, sporulation and pathogenicity of transformed and field strains carrying L412F and L412V mutations

**Figure 5:**
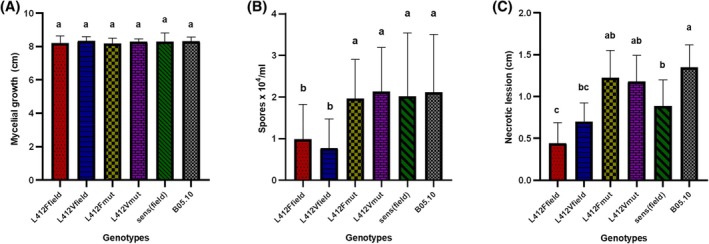
Fitness measurements of *Botrytis cinerea* isolates with different genotypes. (A) Mycelial growth (cm in diameter) of different *B. cinerea* strains after being incubated at 20 °C for 5 days in the dark, on PDA medium. (B) Amount of spores (spores per milliliters) produced by different *B. cinerea* genotypes after being incubated at 20 °C for 14 days in the dark in PDA medium. Values were logarithm transformed before analysis. (C) Diameter of necrotic lesions (cm) on bean plant cotyledons caused by different genotypes of *B. cinerea* strains. Bean plants had their cotyledons inoculated with conidial suspensions of *B. cinerea* and were inoculated at 22 °C with 100% relative humidity for 5 days. Six cotyledons were infected per fungal genotype. L412Ffield and L412Vfield refers to field isolates carrying the indicated mutations, whereas L412Fmut and L412Vmut denote mutants derived from the B05.10 strain. Sens(field) refers to AP‐sensitive field strains. Five isolates were used per genotype, each tested in three technical replicates. All experiments were repeated three times using independent biological material. Different letters indicate statistically significant differences according to Tukey's test (*α* = 0.05).

The mycelial growth of all tested genotypes, including transformed and field L412F/V strains, sensitive field strains and the model B05.10 strain, did not differ significantly, with colony diameters ranging from 7.95 to 8.11 cm, suggesting that the L412F/V mutations do not affect the ability of *B. cinerea* isolates possessing them to grow in PDA medium (Fig. 5(A)).

Regarding sporulation, there was no significant difference between the L412F and L412V mutants, nor among the mutants and B05.10, suggesting that these two mutations do not seem to lead to altered sporulation levels. However, AP‐resistant isolates originating from the field displayed lower sporulation values compared to all the other tested genotypes (Fig. 5(B)).

The pathogenicity tests were carried out on the cotyledons of bean plants and all strains caused symptoms. All strains caused symptoms on the bean plants. No significant difference was observed between the pathogenicity of the mutants possessing the L412F or L412V mutations and the B05.10 strain, suggesting that these mutations hardly seem to affect pathogenicity. Field strains proved less fit to induce the same symptoms as the transformed strains or the B05.10, by causing two times smaller lesion (Fig. 5(C)).

## 4 DISCUSSION

Several fungicides are currently used in agricultural practice, yet their specific mode of action remain unidentified, with AP fungicides falling into this category. While originally thought to inhibit methionine biosynthesis, new evidence supports probable involvement in mitochondrial processes.^6^ Fungal populations of *B. cinerea* have developed resistance to this fungicide class, especially in heavily treated crops.^14^, ^16^, ^43^, ^44^ Apart from proposing a new mode of action, Mosbach *et al*.^6^ also identified genes related to AP resistance, as well as the most frequent mutations leading to it. Since resistance management in the field is deemed crucially important, early detection of resistance and fitness assessment of resistance conferring mutations are of relevance.

The current study aimed to develop a rapid method for the identification of some of the most common target genes’ mutations associated with resistance to AP fungicides in *B. cinerea* populations and to characterize resistant mutants generated by using the CRISPR/Cas9 gene editing method. T‐ARMS PCR is a modified multiplex PCR used for SNP detection in a single reaction, employing four primers: two outer primers and two allele‐specific inner ones. While its optimization is quite challenging, it provides an easy, cost‐effective and rapid way of detecting allele‐specific modifications. As a molecular diagnostic tool, its usage ranges from various fields, like human, mammalian, marine, to fungal biology.^18^, ^45^, ^46^, ^47^ When applied to diploid organisms, it usually aims to detect a desired SNP, with one of the inner primers binding only to the mutated allele, whereas the other inner primer binds to the WT allele. Based on the different length of the specific generated PCR products, a simple gel electrophoresis can detect the presence or absence of the mutated allele, while also providing information about homozygosis or heterozygosis. In this study, we took advantage of *B. cinerea* haploid state in nature and designed both inner primers to detect two distinct AP resistance‐conferring mutations. These mutations result from different nucleotide substitutions in the same codon, namely L412F and L412V. Despite *B. cinerea* haploidy, its conidia tend to be multinucleate^48^ meaning that different alleles could still be present in the same cell because of heterocaryosis. While the generated T‐ARMS PCR tool is not designed to identify the WT allele or provide a measure of quantification of resistance alleles, it can be used quite efficiently to detect the presence of these two AP resistance‐conferring mutations.

Several studies have used digestion techniques to further identify mutations, for example, the RFLP technique.^49^, ^50^ In our study, samples yielding a single PCR band resulting from the outer primers' binding were further digested using MlyI to allow discrimination of the products, specifically between G408V and WT alleles. However, here we report for the first time the combined use of T‐ARMS PCR followed by enzymic digestion of the same PCR products in *B. cinerea*, which further reduces both time and cost. By testing the detection tool, the results showed that L412F was by far the most frequent AP‐resistance mutation followed by L412V and G408V. These results are in accordance with Mosbach *et al*.,^6^ who were the first to propose and find these mutations in the field. In addition, the co‐occurrence of different mutations in *Bcpos5* (e.g., G408V and L412F) has not been reported or appears to be rare. However, in our results E407K mutation in *Bcmdl1* was found at a percentage of just 5% whereas this specific mutation was found predominant in Chinese populations with frequencies of almost 65%.^27^ The variability of mutations conferring resistance to AP fungicides and their different frequencies within the field populations in different geographic regions might be related to different patterns of use of the different AP active substances used in each region. However, further studies are required to confirm this hypothesis.

The generation of mutants is a crucial step in studies involving functional analyses and impacts of identified mutations. The first successful attempts of targeted mutagenesis combined the use of selection markers with homologous recombination.^51^ Common resistance cassettes (i.e., hygromycin, fenhexamid, nourseothricin) are widely used as markers for the selection of potential successful transformants. However, small scale genetic manipulations like point mutation replacements are limited by these approaches. CRISPR/Cas is an innovative method that has been applied in various aspects of gene editing. Its basic principle involves the generation of a targeted DNA DSB, compelling the organism to repair the damage by using the two major DNA repair mechanisms: NHEJ and HR.^52^ The system consists of the Cas9 endonuclease and the sgRNA, formed together in a RNP complex. Newer advances have utilized the *in vitro* assembly of the RNP, offering numerous advantages.^30^ In addition, Leisen *et al*.^34^ recently developed a potent genome editing tool for the markerless transformation of *B. cinerea*. Briefly, purified RNP along with DNA donor template are added to the protoplasts. In our study, we took advantage of this well adapted system to create mutants possessing point mutations leading to L412F and L412V substitutions in the *Bcpos5* gene. L412F and L412V mutations were the target of our efforts since these are the two most common mutations associated with resistance to AP fungicides in the European populations.^6^, ^16^ The standard protoplast transformation protocol was used,^41^ but since the desired mutations lead to AP resistance, no helper plasmid was required and the selection was directly performed on cyprodinil‐amended plates. While the generation of L412V mutants succeeded on the first try, L412F required an additional overlay with substrate with no selection medium before applying the selection pressure. This is in accordance with Mosbach *et al*.^6^ indicating that the resistance phenotype might require additional time to be fully expressed. The major limitation of the CRISPR/Cas editing tool is the potential off‐target activity, meaning that the system may also generate additional DNA breaks in unwanted genomic loci, because of similarities between the sequence of the sgRNA and the whole genome. However, deliverance of *in vitro* assembled RNP instead of transgenic expression of Cas9 and sgRNA lowers the risk of off‐target activity because of the limited half‐life of the RNP complex in the cell. Furthermore, online tools exist, for example, CHOPCHOP, Broad Institute and so forth, that aid in designing sgRNA with increased level of efficiency and specificity. These tools along with performing BLAST of the designed sgRNA sequence to the *B. cinerea* genome can significantly lower the risk of off‐target modifications.^34^

Fungicide resistance in plant pathogens is almost entirely attributed to point mutations in corresponding gene targets. However, these changes occurring in functional domains of proteins may lead to fitness cost, meaning that strains possessing these mutations are outperformed by WT strains in the absence of a selection pressure like fungicide applications. Fungicide resistance in *B. cinerea* is well studied, and so are the potential fitness penalties of mutations conferring resistance. In previous studies, isolates harboring different point mutations like H272R/Y/L, N230I, and P225F in *sdhB* gene displayed both moderate to high resistance to at least some succinate dehydrogenase inhibitors (SDHIs) and reduced fitness.^23^, ^42^ Moreover, both laboratory and field strains resistant to fenhexamid displayed lower levels of conidia and sclerotia production.^22^, ^43^, ^53^ Similarly, *B. cinerea* isolates highly resistant to fludioxonil have been associated with fitness costs associated with increased osmotic sensitivity and they are not found in the field. In practice resistance to fludioxonil primarily comes from drug efflux mechanisms.^54^, ^55^, ^56^ However, no fitness costs were observed in isolates possessing the G143A mutation in *cytb*.^42^ Regarding AP fungicides, no direct fitness impairment was detected in cyprodinil‐resistant isolates.^25^, ^27^ Nevertheless, most of the studies were conducted on field populations making it difficult to exclude the potential effect of other mutations in different genomic regions apart from resistance related loci. Fan *et al*.^27^ generated *B. cinerea* mutants possessing the E407K mutation in *Bcmdl1* gene that confers resistance to AP fungicides. At the same time, no significant differences were observed between these laboratory mutants and the WTs, suggesting that the E407K substitution does not affect overall fitness.^27^ In our study, the unknown genetic background of the field strains appears to strongly influence their fitness, however, when comparing the transformed strains with B05.10, it is becoming evident that L412F and L412V substitutions do not affect fitness parameters like *in vitro* mycelial growth, sporulation and, *in planta* virulence since laboratory mutants possessing L412F and L412V mutations did not display significant differences compared to B05.10 parent isolate. It should be noted that field strains included in this study were characterized exclusively for AP resistance‐associated phenotypes, whereas other resistance mechanisms (e.g., multi‐drug resistance) were not assessed. For example, in our study field‐derived AP‐resistant isolates exhibited reduced sporulation capacity relative to their mutant counterparts. This phenotype may reflect the accumulation of additional resistance‐associated mutations to other fungicide classes or, more broadly, the presence of genomic alterations that impose a fitness cost affecting the sporulation process. While it may seem that AP resistance‐conferring mutations do not inhibit the basic biological processes of the fungus, there are countless aspects that may contribute to the overall fitness of the pathogen. For example, conidia and sclerotia production, mycelial growth and sporulation *in vitro*, and pathogenicity are some important and commonly studied fitness parameters. However, in reality, the situation in the field is very dynamic and competitive, and numerous other aspects influence fitness, like inter‐ and intra‐species competition, signaling, energy efficiency, overwintering, and so forth. While at a first glance these mutations do not seem to affect some important features of the fungus' lifestyle, they may lead to reduced performance under field conditions in the absence of selection pressure. The most dominant AP resistance‐conferring mutation in China seems to be E407K, while in Europe L412F seems to dominate the fungal population (this study),^6^, ^16^ It is noteworthy though that both mutations are present in both continents.^6^, ^16^, ^27^ It could be assumed that there is no fitness difference between these two mutations since they do not seem to follow a pattern of prevalence worldwide. Since AP fungicide characteristics like mode of action and resistance mutations have only recently been proposed,^6^ it would be beneficial to study the mutations' presence worldwide and their impact on different steps of the life cycle of the pathogen.

In summary, this study successfully developed a rapid and cost‐effective molecular tool combining T‐ARMS PCR and enzymatic digestion for the detection of key AP resistance‐conferring mutations in *B. cinerea*. The application of CRISPR/Cas9 technology enabled the generation of isogenetic mutants, facilitating functional analyses of L412F and L412V mutations. Our findings highlight the predominance of L412F in Greek populations, contrasting with the E407K mutation frequently observed in China, underscoring regional differences in resistance dynamics. Importantly, laboratory mutants exhibited no significant fitness penalties, suggesting these resistance mutations may persist in the absence of fungicide pressure. However, the complexity of pathogen fitness in field conditions warrants further investigation to fully understand the ecological impact of these mutations. Continued surveillance and molecular characterization of resistance mutations worldwide will be essential for informed resistance management and sustainable crop protection strategies.

## FUNDING INFORMATION

This work was founded by the European Union‐Next Generation EU, Greece 2.0 National Recovery and Resilience plan. Project title ‘Evidence based management of pesticide resistance in vegetable crops’ (Μ16ΣΥΝ2‐00094).

## CONFLICT OF INTEREST

The authors declare that they have no known competing financial interests or personal relationships that could have appeared to influence the work reported in this article.

## AUTHOR CONTRIBUTIONS

George Sofianos: writing – original draft, visualization, methodology, investigation, formal analysis, data curation, conceptualization. Athanasios Petmezas: methodology, investigation, formal analysis, data curation. Anastasios Samaras: supervision, investigation, formal analysis, data curation, conceptualization. George Karaoglanidis: writing – review and editing, validation, supervision, project administration, funding acquisition, conceptualization.

## Supporting information


**Table S1.** Oligonucleotides used for transformation and validation.

## Data Availability

The data that support the findings of this study are available from the corresponding author upon reasonable request.
