# Supplementary material for: Resistance of Botrytis cinerea to anilinopyrimidine fungicides: A novel ARMS‐PCR method for the detection of Bcpos5 mutations and characterization of resistance using CRISPR/Cas9 editing
Source: Pest Manag Sci. 2026 Apr 28;82(9):8189–98. doi: 10.1002/ps.70864 (PMC13453242; doi:10.1002/ps.70864)
Supplement: Supplementary file 1 [file PS-82-8189-s001.docx]

**Supplementary Table 1**: Oligonucleotides used for transformation and validation

| Primer name | Primer sequence (5’ – 3’) | Usage in this study |
| --- | --- | --- |
| sgRNA *Bcpos5* | AAGCTAATACGACTCACTATAGGTTGGGTTGGAGGCTTGAAGTTTTAGAGCTAGAAATAGCAAG | Oligonucleotides used for the generation and *in vitro* transcription of sgRNA |
| sgRNA constant region | AAAAGCACCGACTCGGTGCCACTTTTTCAAGTTGATAACGGAC  TAGCCTTATTTTAACTTGCTATTTCTAGCTCTAAAAC |  |
| Fw primer 412F/V | GTGGGGGCGTTCCTTGTGTCATGAGAGGGGCCAAAAGTGGCATGAAA  GATGACGACGGTTGGGTTGGAGGCTTGAATGG**C** | Oligonucleotide used for the generation of donor template |
| Rev primer L412F | GCCTTGGTGATAGAATTGATATCCACCATTCAATCGATCTCG  GACATTATTCTTCGCCAAAAGGATGGTTGAACTTCAG***A***AA**G**CCATTCAAGCCTCCAA | Oligonucleotide used for the generation of donor template possessing L412F mutation |
| Rev primer L412V | GCCTTGGTGATAGAATTGATATCCACCATTCAATCGATCTCGGACATTA  TTCTTCGCCAAAAGGATGGTTGAACTTCAGCA***C*G**CCATTCAAGCCTCCAA | Oligonucleotide used for the generation of donor template possessing L412V mutation |
| MDL_Fw | GAGGTGCCGGATTTACAGCT | Used for PCR amplification and sequencing of part of *Bcmdl1* gene |
| MDL_RV | GTCGAGTTGGGTAGGCGAAA |  |

Bold characters indicate the inserted nucleotide changes.

Italicized characters denote the intended nucleotide substitutions resulting in the amino acid replacements L412F and L412V, underlined characters indicate silent mutations introduced in the PAM sequence.
